# Supplementary material for: Novel Synthesis Route of Plasmonic CuS Quantum Dots as Efficient Co-Catalysts to TiO2/Ti for Light-Assisted Water Splitting
Source: Nanomaterials (Basel). 2024 Sep 30;14(19):1581. doi: 10.3390/nano14191581 (PMC11478289; doi:10.3390/nano14191581)
Supplement: Supplementary file 1 [file nanomaterials-14-01581-s001.zip › nanomaterials-3192676-supplementary.pdf]

Novel synthesis route of plasmonic CuS quantum dots as efficient co-catalysts to TiO_2/Ti for light assisted water splitting

Larissa Chaperman,[1] Samiha Chaguetmi,[2] Bingbing Deng,[1] Sarra Gam-Derrouich,[1] Sophie Nowak,[1] Fayna Mammeri,[1] Souad Ammar [1,*]

[1] Université Paris Cité, CNRS UMR-7086, ITODYS, 75205 Paris, France
[2] Université 20-Août-1955-Skikda, Faculté des Sciences, 21000 Skikda, Algeria

(*) Corresponding author : souad.ammar-merah@u-paris.fr

Supporting information

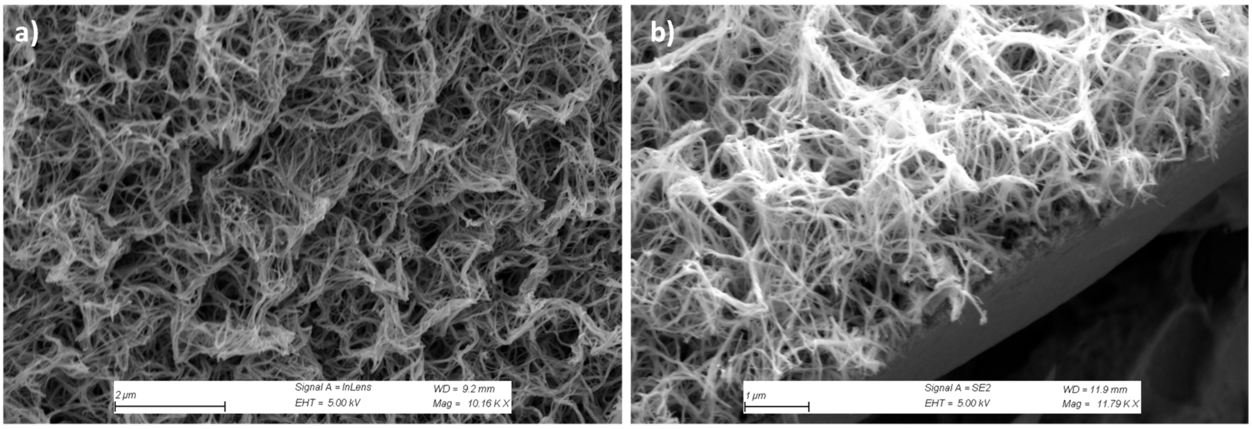

Figure S1. SEM **a)** top view and **b)** cross view of the as prepared TiO_2/Ti sheet confirming the 1D morphology of the formed titania, covering all the Ti substrate surface.

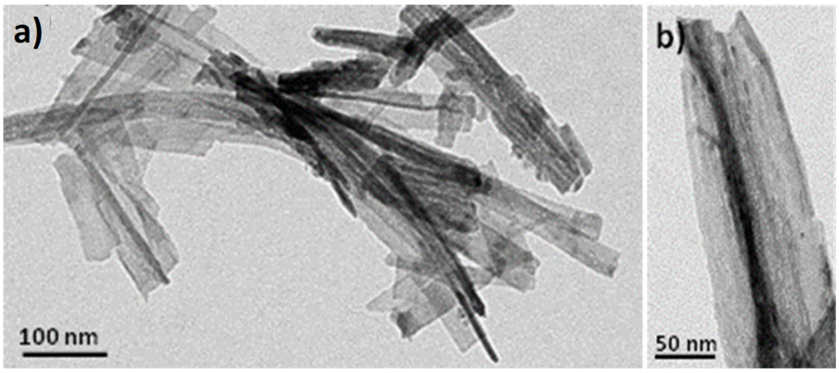

Figure S2. TEM micrographs recorded on **a)** an assembly and **b)** an individual representative TiO_2 NFs separated by sonication from the as-produced TiO_2/Ti sheets in an ethanolic solution. Titania fibers appear as veils folded on themselves.

CuS reference	MW Power (W)	MW Heating time (min)	Sulfur source
CuS-ThU-1200-1	1200	1	ThU
CuS-ThU-1200-3	1200	3	
CuS-ThU-200-25	200	25	
CuS-TAA-1200-1	1200	1	TAA
CuS-TAA-1200-3	1200	3	
CuS-TAA-200-25	200	25	

Table S1. List of prepared CuS samples and their main synthesis MW-assisted polyol synthesis

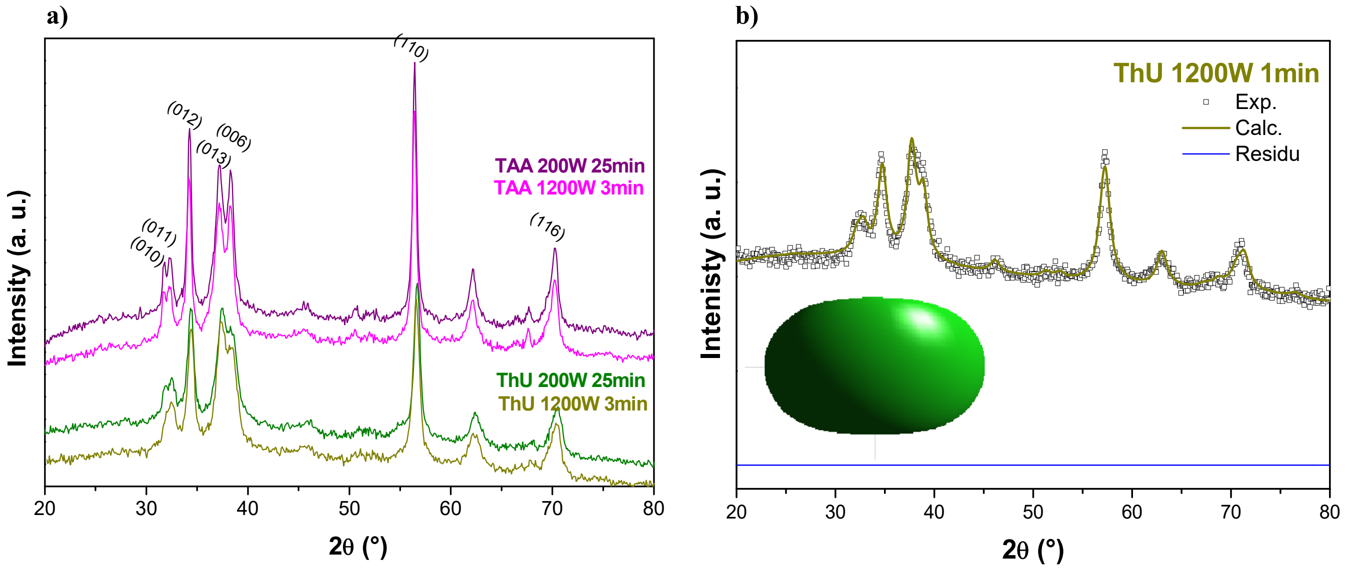

Figure S3. **a)** XRD patterns of CuS particles prepared using TAA (up) and ThU (bottom) reagents while applying a microwave heating power of 200 and 1200 W for a total reaction time of 25 and 3 min, respectively. **b)** Results of Rietveld refinements (using MAUD software [1]performed on the XRD pattern of CuS-ThU-1200-1 particles to illustrate the quality of the fits: the experimental pattern (black scatter) and the calculated one (green line) are perfectly superposed with a residue curve, defined as the difference between the experimental and calculated diffractograms, close to zero (blue line). The inferred crystallite shape is also given for information.

	R_{wp} (%)	R_B (%)	R_{exp} (%)	a (Å) ± 0.005	c (Å) ± 0.005	$L_{[006]}$ (nm) ± 0.5	$L_{[100]}$ (nm) ± 0.5
**TAA 200W 25min**	1.57	1.10	0,79	3.789	16,386	12.2	19.1
**TAA 1200W 3min**	1.64	1.13	0.83	3.789	16,366	12.1	14.9
**TAA 1200W 1min**	1.70	1.33	1.13	3.786	16,306	10.1	13.0
**ThU 200W 25min**	1.52	1,06	0.82	3.787	16,324	9.9	11.6
**ThU 1200W 3min**	1.40	1.00	0.80	3.791	16,386	9.9	10.8
**ThU 1200W 1min**	1.66	1.29	1.15	3.780	16,309	7.8	7.7
**Covellite CuS standard (ICDD N°98-004-1975)**				3.796	16.360	-	-

Table S2. Main Rietveld refined structural parameters and their related reliability fit factors. The overall fit quality is described by a weighted profile (R_{wp}), expected profile (R_{exp}) and Bragg R-value (R_B) close to 1, which is the case here.

[1] L. Lutterotti, S. Matthies, H. R. Wenk, MAUD: a friendly Java program for material analysis using diffraction, *IUCr: Newsletter of the Commission on Powder diffraction*, **1999**, *21*, 14-15.

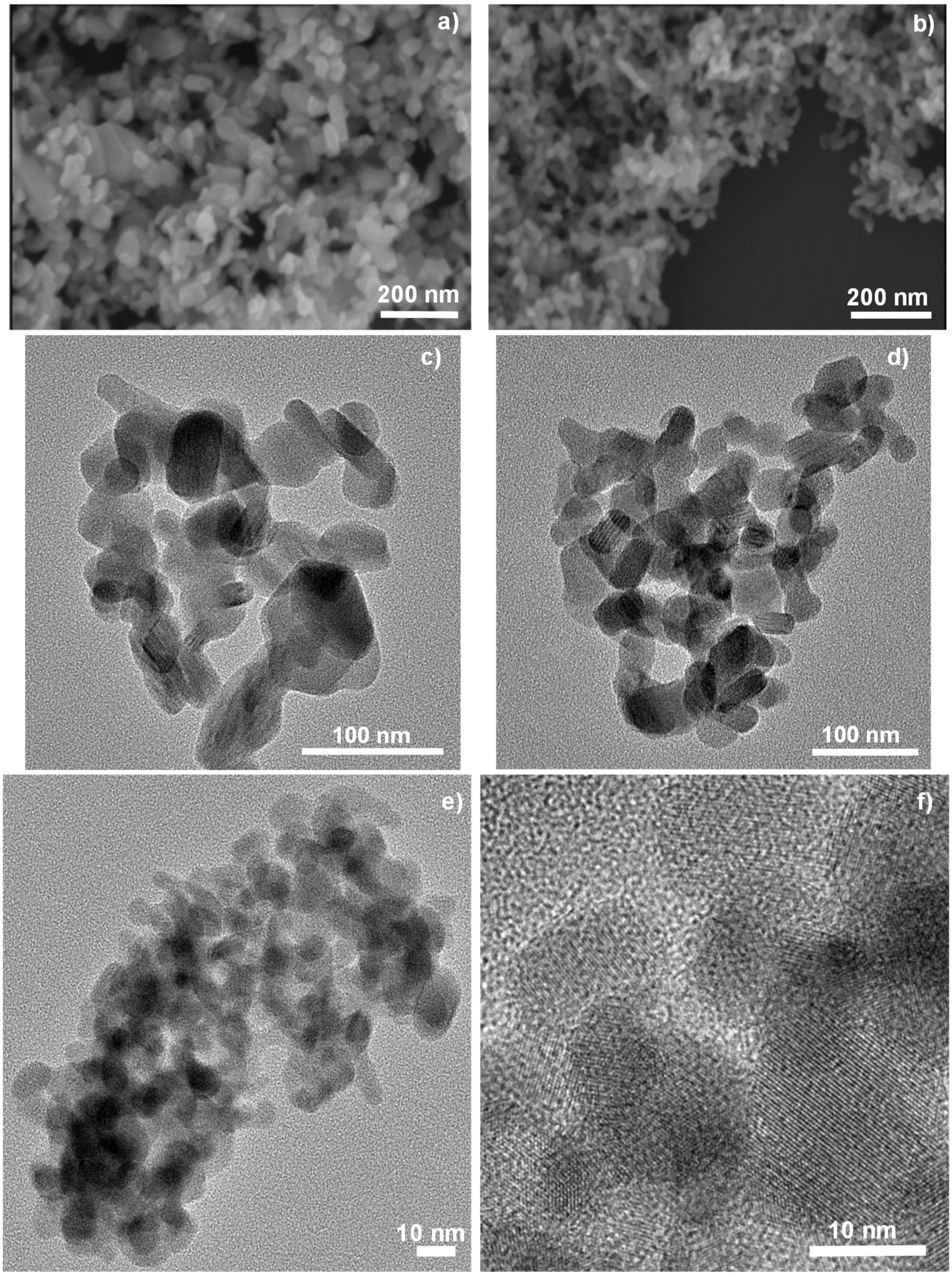

Figure S4. SEM images recorded on **a)** CuS-TAA-200-25 and **b)** CuS-ThU-200-25 particles. TEM images of **c)** CuS-TAA61200-3 and **d)** CuS-1200-ThU-1200-3 particles. **e)** TEM micrograph of CuS-ThU-1200-1 particles and **f)** HRTEM image of some representative CuS-ThU-1200-1 particles.

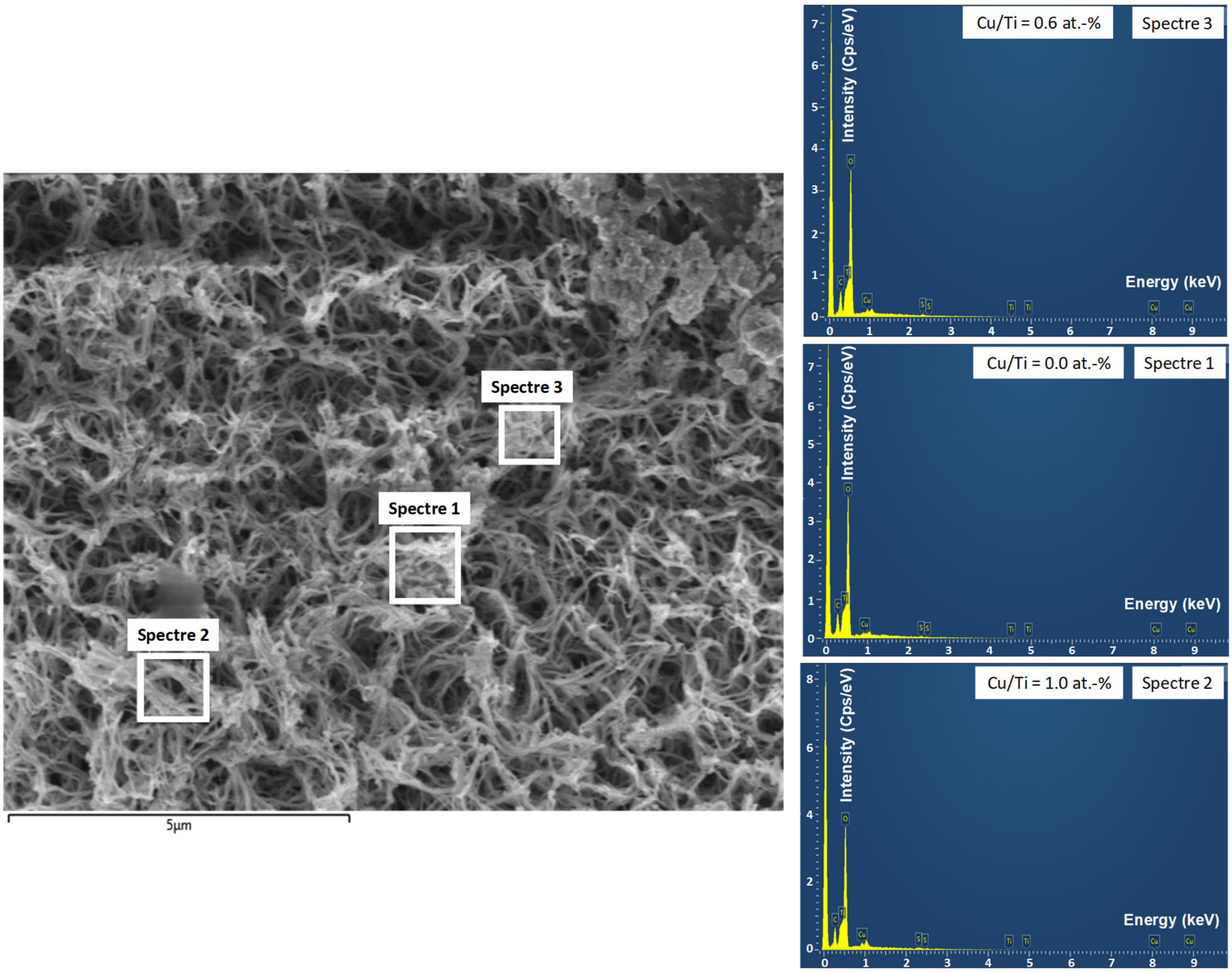

Figure S5. SEM-EDS analysis of CuS-TiO_2/Ti, focusing on TiO_2 fibers nodes, where CuS particles seem to accumulate, leading to an average Cu/Ti atomic ratio of 0.5 at.-%.

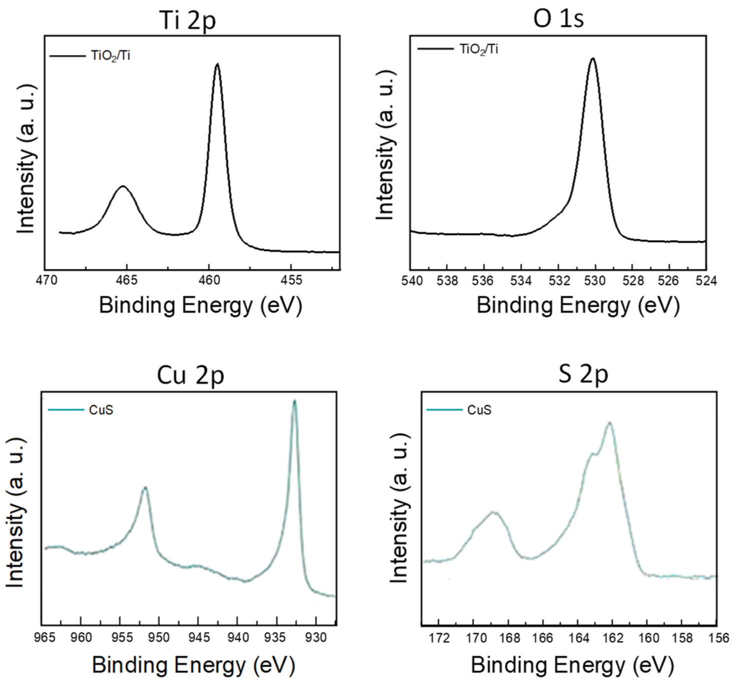

Figure S6. Ti 2p and O 1s high resolution XPS spectra recorded on pristine TiO_2/Ti (black) and Cu2P and S 2p high resolution XPS spectra recorded on pristine CuS (blue-green line).

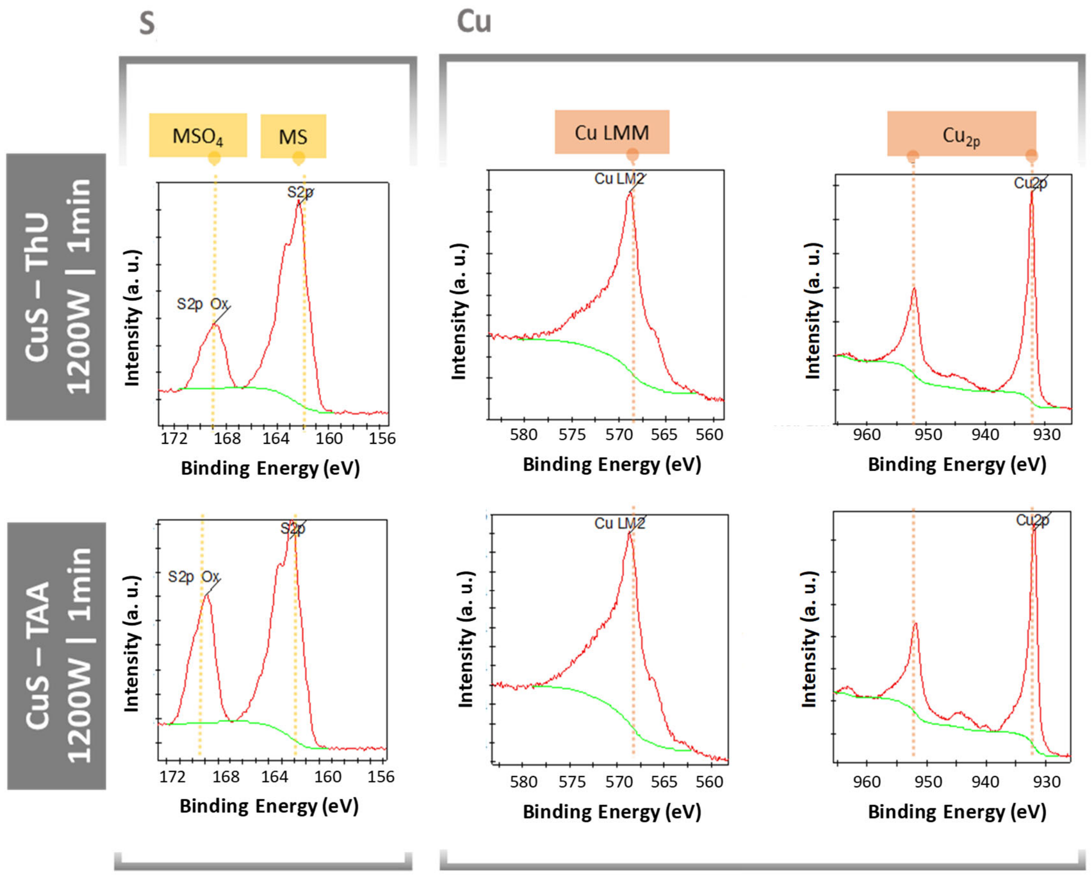

Figure S7. S 2p, Cu 2p and Cu LMM high resolution XPS spectra recorded on CuS NPs produced using ThU or TAA sulfur source for a microwave heating power of 1200 W along 1 min of reaction time.

Composite	Synthesis method	Electrolyte	Light source	Photocurrent density (mA/cm^2)	Ref
CuS/TiO_2 nanocomposites	Co-precipitation	0.1 M Na_2SO_4	UV-LED lamp (25 W, 365 nm)	0.2 (at 0.6V *vs.* Ag/AgCl)	[2]
CuS/TiO_2 nanocomposites	Hydrothermal	0.1 M Na_2SO_4	UV-LED lamp (25 W, 365 nm)	0.17 at 0.6V *vs.* Ag/AgCl	[2]
CuS nanoflakes/TiO_2 Nanospindles	hydrothermal and solution-based process	0.5 M Na_2SO_4	Xe arc lamp (300 W)	0.12 (at 0.6V *vs.* SCE)	[3]
TiO_2/CuS nanowire array heterostructures	SILAR method	1 M Na_2SO_4	AM 1.5G sunlight simulator (100 mW/cm^2)	0.063 (at 0V *vs.* Ag/AgCl)	[4]
TiO_2@CuS double-shell nanoboxes	constructed via a multistep control strategy	0.35 M Na_2S + 0.25 M Na_2SO_3	chopped illumination *of* simulated sunlight	0.032 (at 0V *vs.* Ag/AgCl)	[5]
TiO_2/CuS core-shell nanorods	CuS(*n*) shell produced by ion exchange from ZnS(*n*) counterparts prepared by SILAR route	–	AM 1.5G sunlight simulator (100 mW/cm^2)	3.32 (at 0V *vs.* Ag/AgCl)	[6]
CuS-TiO_2 composite	Electrophoretic deposition	0.50 M CH_3OH as electroactive species in $Na_2CO_3/NaHCO_3$ (pH = 9.5)	Visible light irradiation	0.3 (at 0V *vs.* SCE)	[7]
CuS-TiO_2 core-shell structure	Hydrothermal method	0.1 M PBS and 0.1 M KCl	Xe lamp with a 400 nm cut-off filter	0.0054 (at 0.1V *vs.* Ag/AgCl)	[8]
CuS/TiO_2(NTs)	SILAR method	0.1 M PBS and 10 mM glucose	Xe lamp (400 W)	0.0118 (at 0V *vs.* Ag/AgCl)	[9]
CuS/TiO_2	Wet impregnation	3.5 wt.-% NaCl in water	Hg-Xe lamp UV with a 365 nm band-pass filter (1 mW/cm^2)	9.3 (at 0V *vs.* Ag/AgCl)	[10]
CuS-TiO_2	*in situ* chemical bath deposition	–	AM 1.5G sunlight simulator (100 mW/cm^2)	4.5 (at 1V *vs.* Ag/AgCl)	[11]
CuS-TiO_2(NFs)	Ethanolic impregnation	0.5 M Na_2SO_4 (pH = 7.0)	Xeon lamp (150 W)	0.030 (at 0V *vs.* SCE) 0.122 (at 1.23V *vs.* SCE)	(*)

(*) This work

Table S3. Comparison of the PEC performances of our engineered photoanode with those of CuS-TiO_2 based literature.

[2] R. A. El-Gendy, H. M. El-Bery, M. Farrag, D. M. Fouad, Metal chalcogenides (CuS or MoS_2)-modified TiO_2 as highly efficient bifunctional photocatalyst nanocomposites for green H_2 generation and dye degradation, *Sci. Rep.*, **2023**, *13*, 7994.

[3] M. Chandra, K. Bhunia, D. Pradhan, Controlled Synthesis of CuS/TiO_2 heterostructured nanocomposites for enhanced photocatalytic hydrogen generation through water splitting, *Inorg. Chem.*, **2018**, *57*, 4524–4533.

[4] S. Jia, X. Li, B. Zhang, J. Yang, S. Zhang, S. Li, Z. Zhang, TiO_2/CuS heterostructure nanowire array photoanodes toward water oxidation: The role of CuS, *Appl. Surf. Sci.*, **2019**, *463*, 829-837.

[5] B. Yu, F. Meng, T. Zhou, A. Fan, M. W. Khan, H. Wu, X. Liu, Construction of hollow TiO_2/CuS nanoboxes for boosting full-spectrum driven photocatalytic hydrogen evolution and environmental remediation, *Ceram. Int.*, **2021**, *47*, 8849-8858.

[6] X. Zhao, K. Zhao, J. Su, L. Sun, TiO_2/CuS core-shell nanorod arrays with aging-induced photoelectric conversion enhancement effect, *Electrochem. Commun.*, 2020, *111*, 106648.

[7] Z. Huang, Z. X. Wen, X. Xiao, Photoelectrochemical Properties of CuS-TiO_2 Composite Coating Electrode and Its Preparation via Electrophoretic Deposition, *J. Electrochem. Soc.*, **2011**, *158*, H1247–H1251.

[8] Y. Tang, Y. Chai, X. Liu, L. Li, L. Yang, P. Liu, Y. Zhou, H. Ju, Y. Cheng, A photoelectrochemical aptasensor constructed with core-shell CuS-TiO_2 heterostructure for detection of microcystin-LR, Biosensors Bioelec., **2018**, *117*, 224–231.

[9] Y. Wang, L. Bai, Y. Wang, D. Qina, D. Shan, X. Lu, Ternary nanocomposites of Au/CuS/TiO_2 for ultrasensitive photoelectrochemical non-enzymatic glucose sensor, *Analyst*, **2018**, *143*, 1699–1704.

[10] P. Ngaotrakanwiwat, P. Heawphet, P. Rangsunvigit, Enhancement of photoelectrochemical cathodic protection of copper in Marine Condition by Cu-Doped TiO_2, *Catalysts*, **2020**, *10*, 146.

[11] W. Liu, H. Ji, J. Wang, X. Zheng, J. Lai, J. Ji, T. Li, Y. Ma, H. Li, S. Zhao, Z. Jin, Synthesis and Photo-Response of CuS thin films by an in situ multi-deposition process at room temperature: A Facile & eco-friendly approach, *NANO: Brief Rep. Rev.*, **2015**, *10*, 1550032.
